# Next-Generation Remote Sensing Data at Multiple Spatial Scales Improves Understanding of Habitat Selection by a Small Mammal

**DOI:** 10.3390/ani14223175

**Published:** 2024-11-06

**Authors:** Catherine F. Frock, L. Mike Conner, Robert A. McCleery

**Affiliations:** 1 Department of Wildlife Ecology and Conservation, University of Florida, 314 Newins-Ziegler Hall, P.O. Box 110430, Gainesville, FL 32611, USA; frock@uwalumni.com; 2 The Jones Center at Ichauway, Wildlife Ecology Lab, 3988 Jones Center Drive, Newton, GA 39870, USA; mike.conner@jonesctr.org

**Keywords:** animal tracking, habitat suitability model, multi-scale, global positioning system, fox squirrel, *Sciurus niger*, resource selection

## Abstract

Recent advances in optical remote sensing (RS) technology in combination with lightweight Global Positioning System (GPS) tracking devices now make analyzing the multi-scale habitat selection (HS) of small mammals < 2 kg possible. However, there have been relatively few multi-scale HS studies integrating fine-scale RS data with data-rich, GPS-derived movement data from small mammals. This is critical because small mammals commonly select habitat features across multiple scales. To address this gap, we investigated the HS of a small mammal, fox squirrels (*Sciurus niger*), which are known to cover relatively large areas and select fine-scale environmental features. We specifically asked the following questions: (1) Do next-generation RS variables improve HS models at single spatial scales? (2) Do multi-scale HS models improve upon those at single spatial scales? Using data from 45 individuals, we constructed HS models at three spatial scales: 4 ha (210 m × 210 m), 0.09 ha (30 m × 30 m), and 0.01 ha (10 m × 10 m) using traditional and next-generation RS data. The 4-ha model, using traditional and next-generation RS data, produced the best single-scale model, explaining 58% of the variations in HS. However, the multi-scale model provided the most informative model, explaining 68% of the variations in HS. Our models provide evidence for the value of next-generation RS data when quantifying HS and additional support for the idea of studying HS at multiple spatial scales.

## Simple Summary

Habitat selection studies are important in wildlife ecology and conservation. Habitat selection is often quantified using tracking devices attached to animals to determine the animals’ locations. Environmental variables at these locations are then assigned to the location and subsequently used to infer habitat selection using a variety of statistical approaches. Until recently, the spatial scale of remote sensing data and the weight of tracking devices for small mammals have been insufficient for use on small mammals. Now, GPS tags are sufficiently small enough for use on many small terrestrial vertebrates, and remotely sensed data are available at fine spatial scales. We found that the combination of small GPS trackers and high-resolution spatial data allowed for an improved understanding of resource selection in fox squirrels.

## 1. Introduction

Animals often select habitats on multiple scales, selecting characteristics at broader home-range scales that do not necessarily mirror some of the finer-scale features they prefer [1]. To capture the patterns of selection across broad scales, traditional studies of animal habitat selection have often included categorical measures of land cover and topography metrics [1,2]. This has changed over the last several decades, as the availability of remotely sensed (RS) data from satellite-based platforms has greatly improved the resolution (>30 × 30 m) at which researchers can measure habitat characteristics over broad spatial extents. This finer-scale RS data have been useful for understanding animal habitatselection [1,3], particularly with large animals, which often select habitats based on coarser scales [1,4]. However, it has been less valuable for understanding the habitat selection of small-bodied animals (<2 kg), which often respond to environmental variations at scales finer than 30 × 30 [1]. In fact, until recently, the resolution of RS data [2,4] and size of tracking devices [5] has constrained researchers’ ability to study smaller animals’ habitat selection over expansive areas. Today, both the resolution of RS and the size of GPS tags have improved to accommodate smaller animals, allowing researchers to match the grain of RS with GPS datasets for many small-bodied animals [5].

As the size and weight of GPS tags have decreased [5], RS products have become more cost-effective, available at finer scales, and more advanced. Some airborne and satellite platforms now utilize light detection and ranging (LiDAR), which measures features in three spatial dimensions [6,7]. Further, advances in RS data can now provide a wider suite of next-generation RS products, such as biomass (BIOM), canopy height models (CHMs), and numerous vegetation-related metrics (e.g., fraction of photosynthetically active radiation (fPAR), Leaf Area Index (LAI), Moisture Stress Index (MSI), and Normalized Difference Vegetation Index (NDVI)), that should enhance our understanding of an animal’s habitat needs. However, despite their tremendous potential in regard to better understanding animal ecology, these novel next-generation RS products have not received widespread use in animal ecology. Specifically, there have been relatively few multi-scale HS studies that have integrated fine-scale RS data with data-rich, GPS-derived movement data from small mammals [8]. However, we have seen an increase in small-mammal studies that link fine-scale RS data that was collected across broad scales with occurrence data [9,10] and VHF telemetry data [1]. This is critical because small mammals are more likely to perceive and respond to environmental variations at finer scales [1] than larger mammals.

To explore the potential of new technologies to better understand small-mammal habitat selection, using GPS tags, we evaluated the utility of traditional and next-generation RS data to understand fox squirrel (*Sciurus niger*) habitat selection on multiple scales. Fox squirrels were an excellent model to use to test these technologies because of their propensity to rapidly cover long distances (>500 m in 1 h) [11] while selecting specific trees for foraging and nesting [12]. At single spatial scales, we expected the addition of next-generation variables would improve upon models based on traditional variables. Also, we expected variable importance to vary among spatial scales because many animals select habitat variables differently based on spatial scales. We then determined whether RS variables at multiple spatial scales further improved our understanding of habitat selection relative to single-scale models. We expected that the multi-scale model would be more informative than any single-scale model because of fox squirrels selecting based on different variables at multiple spatial scales.

## 2. Materials and Methods

### 2.1. Study Area

**Figure 1:**
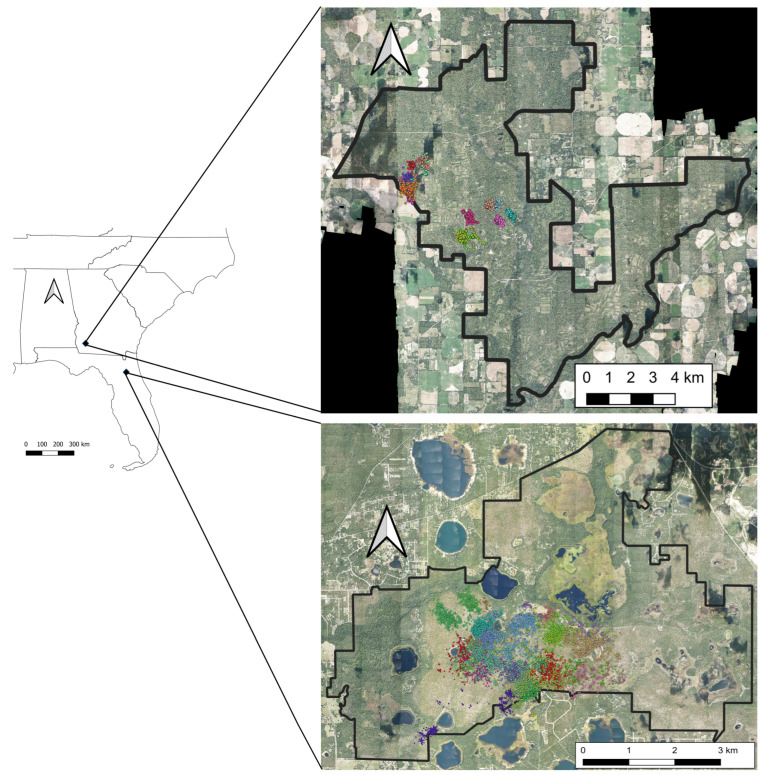
Study area locations. The Jones Center at Ichauway (**Top**) in Georgia and the Ordway-Swisher Biological Station (**Bottom**) in Florida. Fox squirrel GPS data points were colored coded by squirrel ID. Imagery accessed from https://data.neonscience.org/data-products/DP3.30010.001/RELEASE-2024, accessed on 17 October 2024.

We conducted our study on fox squirrels in 2016 and 2017 at the Jones Center at Ichauway (JCI) in Georgia and the Ordway-Swisher Biological Station (OSBS) in Florida in the southeastern United States (Figure 1). Both study areas included forests dominated by longleaf pine (*Pinus palustris*) with other conifers (*Pinus* spp.) and mixed hardwoods interspersed with open herbaceous understories that were maintained using prescribed fire. The regional climate is characterized by annual average highs of 25 and 27 °C (the JCI and OSBS, respectively) and annual average lows of 12 and 14 °C (the JCI and OSBS, respectively). The average annual precipitation is 141.0 and 120.4 cm (the JCI and OSBS, www.usclimatedata.com/climate/newton/georgia/united-states/usga1073 and www.usclimatedata.com/climate/gainesville/florida/united-states/usfl0163, respectively; both accessed 31 October 2021). Elevations throughout study sites are <100 m above sea level.

### 2.2. Study Species

Fox squirrels commonly occur in mature longleaf pine forests with open understories [13]. Fox squirrels take refuge overnight in tree cavities or leaf nests constructed in trees [12,14]. Large trees are important for refuge sites [15], are better for resting and nesting than smaller trees, are more likely to have cavities that fox squirrels can use, and may produce more food resources (e.g., cones with seeds) [16]. At the JCI, seasonal fox squirrel home ranges using VHF tracking averaged 35.8 ha for males and 13.4 ha for females [17]. Average home ranges of up to 53 ha (males and females combined) have been measured using VHF technology [18]. Our GPS tracking suggests home ranges may be larger than previously believed (mean 69 ha, males and females combined (unpublished)), and fox squirrels can traverse their home range diameter in as little as 1 h [11].

### 2.3. Animal Location Data

We trapped fox squirrels opportunistically using wooden box traps baited with corn and/or pecans between March 2016 and December 2016 at the JCI and March 2016 and June 2017 at the OSBS. We fitted individuals > 800 g with a LiteTrack RF-30 GPS/VHF collar (Lotek Wireless, Inc., Newmarket, ON, Canada) and ensured the collar weight was <5% of the animal’s body mass. We programmed collars to record 7 to 11 locations per 24 h period, including 1 at night (0200 h EST) when fox squirrels are in their nests. We retrieved data stored on collars via recapture or remote Ultra High Frequency (UHF) download. We then filtered the data to exclude erroneous locations and only retained locations with a dilution of precision values of 1, indicating high accuracy.

### 2.4. Environmental Data

To measure fox squirrel habitat selection, we used traditional RS variables known to be important to fox squirrels, as well as next-generation RS variables that may influence habitat selection through their links with food resources, refuge use, space use, and/or habitat structure (Table 1). First, we obtained 30 × 30 m rasters of tree canopy cover and 12 land cover classes from the National Land Cover Database (NLCD), collected in 2016. These variables are known predictors of fox squirrel habitat use [13,19,20,21]. Next, we obtained 1 × 1 m rasters of 8 variables collected by the National Ecological Observatory Network’s (NEON) Airborne Observation Platform (AOP). NEON RS data are widely recognized for their high quality, emphasizing data accuracy through stringent quality control processes, such as frequent calibrations, consistent data collection methods, and comprehensive metadata documentation [22,23]. Using data collected in September 2017, we used measures of aspect, BIOM, CHMs, fPAR, LAIs, MSIs, NDVIs, and slope [24]. We chose the CHM because of its documented importance to fox squirrels [25]. We chose aspect and slope because of their importance in predicting tree squirrels’ habitat, and we chose the NDVI due to its possible correlation with increased food resources for tree squirrels [26]. We selected the remaining vegetation-related indices (BIOM, fPAR, LAI, and MSI) due to their correlations with environmental characteristics that we know to be or hypothesized to be important to fox squirrels [27].

### 2.5. Data Processing and Analyses

We processed data and conducted all analyses in the R software platform v. 4.40 [28]. To understand how spatial scale of RS variables might predict fox squirrel third-order habitat selection (i.e., habitat use within home ranges [29]), we aggregated all variables to broad (4 ha, 210 × 210 m) and intermediate (0.09 ha, 30 × 30 m) cells and the NEON variables to fine (0.01 ha, 10 × 10 m) cells. Importantly, NLCD variables were not used at the 10 m scale due to product resolution. We chose our largest scale because of known squirrel habitat associations at similar scales (i.e., land cover [20] and tree canopy [30]). We chose the intermediate scale because of historic use and availability of Landsat data at this scale. We chose our fine scale because this closely matched the locational error in our GPS data. We obtained mean values for the numeric environmental layers and modes for the land cover layer using the aggregate function in the *raster* package v3.6.30. We placed aspect into one of four categories representing cardinal directions, north (>315 or <45 degrees), east (45 < 135 degrees), south (135 < 225 degrees), and west (225 < 315 degrees), for use as a categorical predictor variable. We centered and scaled all numeric data using the scale function in the *raster* package v3.6.30 [31].

We treated squirrel GPS locations as a sample of used habitat. We estimated available habitats for each squirrel using 95% minimum convex polygon (MCP) home ranges and the mcp function in the *adehabitatHR* package v0.4.22 [32]. We consider this a biologically based estimate of an individual’s available habitat because fox squirrels can travel the diameter of an average fox squirrel’s home range in as little as 1 h [11]. To represent a sample of available habitat for each squirrel, we selected random points equal to the number of a squirrel’s GPS locations within each squirrel’s 95% MCP using the spsample function in the *sp* package v2.1.4 [33]. We then extracted the environmental data at different spatial scales, as described above, for the used and available points using the extract function in the *raster* package [31].

### 2.6. Statistical Models

To test for differences in variables between used and available points, we modeled the RSF with mixed-effects binary logistic regression models [34]. We included environmental variables and study sites as fixed effects and the squirrel as a random effect to account for variations among squirrels and unequal sample sizes. We standardized continuous variables to facilitate comparison and used Pearson’s correlation coefficient to evaluate the collinearity of the predictor variables. We considered variable pairs with coefficients > 0.7 as highly correlated and removed variables that allowed for the greatest number of non-correlated predictor variables.

**Table 1:** Remotely sensed environmental variables used as predictors in habitat selection models for fox squirrels (*Sciurus niger*) at the Jones Center at Ichauway in Georgia and the Ordway-Swisher Biological Station in Florida in 2016 and 2017.

Remotely Sensed Variable	Data Source: Data Type ^a^	Variable Type	Spatial Scales (m)	Literature Source Used to Justify Variable and/or Spatial Scale	Known Correlations → Link with Fox Squirrel Ecology
Aspect	NEON: LiDAR	Traditional	30, 10	red squirrels [26]	Habitat use ^a^
Biomass (BIOM)	NEON: multispectral	Next-generation	30, 10		Food resources ^a^
Canopy Height Model (CHM)	NEON: LiDAR	Next-generation	30, 10	fox squirrels [30]	Basal area → Refuge, food resources [13,14]
Fraction of Photosynthetically Active Radiation (fPAR)	NEON: multispectral	Next-generation	30, 10		Refuge [12,14]
Land cover	NLCD (Landsat): multispectral	Traditional	210, 30	fox squirrels [13,20,21]	Habitat use
Leaf Area Index (LAI)	NEON: multispectral	Next-generation	30, 10		Time since last burn and frequency of burns [20,21], hardwood basal area → Home range ^a^
Moisture Stress Index (MSI)	NEON: multispectral	Next-generation	30, 10		Vegetation health → Habitat structure [35], food resources ^a^
Normalized Difference Vegetation Index (NDVI)	NEON: multispectral	Next-generation	30, 10	red squirrels [26]	Food resources ^a^
Tree cover	NLCD (Landsat): multispectral	Traditional	210, 30		Refuge use [12,15]
Slope	NEON: LiDAR	Traditional	30, 10	red squirrels [26]	Habitat use

LiDAR = light detection and ranging. NEON = National Ecological Observatory Network. NLCD = National Land Cover Database. ^a^ Hypothesized links (i.e., no supporting literature could be found).

#### 2.6.1. Evaluation of Next-Generation RS Variables

To test whether next-generation RS variables improved our understanding of fox squirrel habitat selection, we first created two coarse-scale models (210 m). This first traditional model used the tree cover data [15], land cover data using Evergreen Forest [13] as a reference category, aspect using north as the reference category, and slope. We then created a next-generation model that included RS variables that have not commonly been used in small-mammal habitat selection studies: BIOM, CHMs, fPAR, LAIs, MSIs, and NDVIs.

We took a similar approach for the intermediate spatial scale (30 m). We again created a traditional model and a next-generation model, as described above, using traditional and next-generation RS variables. However, the BIOM, CHM, and NDVI variables were highly correlated, as were the BIOM and MSI, and the NDVI, fPAR, and LAI variables, so we eliminated BIOM, NDVI, and fPAR from the next-generation model.

At the fine spatial scale (10 m), we excluded land cover and tree cover variables, as NLCD data are unavailable at this scale. Otherwise, we repeated construction of traditional and next-generation models as described above. The BIOM, MSI, and NDVI variables were highly correlated, as well as fPAR and LAI, so we did not include any highly correlated pairs of variables in the construction of the next-generation model. For each spatial scale, after fitting 2 models (one traditional and one using traditional plus next-generation variables), we determined which of the two models was most parsimonious using Akaike’s Information Criterion, corrected for small sample size (AICc) [36], using the AICc function in the *MuMIn* package. To compare the explanatory power of models, we examined marginal pseudo-*r*^2^ values for each model using the delta method from the r.squaredGLMM function in the *MuMIn* package [37]. This value describes the amount of variance explained in each model and is less susceptible to problems associated with larger models [38].

#### 2.6.2. Evaluation of the Multi-Scale Model

To test whether a multi-scale model improved our understanding of fox squirrel habitat selection relative to that of the best single-scale models, we combined only the statistically significant variables from the best fit coarse-, intermediate-, and fine-scale models to create a multi-scale model. None of these variables were highly correlated. After model fitting, we evaluated the parsimony of the best-fit models at each single spatial scale with that of the multi-scale model with AICc using the AICc function in the *MuMIn* package v1.48.4. To compare the explanatory power of the multi-scale model with the single-scale models, we again examined the marginal pseudo-*r*^2^ values for each model using the delta method from the r.squaredGLMM function in the *MuMIn* package [37].

## 3. Results

We collected GPS location data from 45 unique animals (JCI, *n* = 18; OSBS, *n* = 27). We obtained a mean of 913 (±98 SE) locations per animal for a total of 41,104 locations: 7811 at the JCI and 33,293 at the OSBS.

### 3.1. Evaluation of Next-Generation RS Variables

The best model (lowest AIC_c_ and highest pseudo-*r*^2^) of fox squirrel habitat selection at each examined spatial scale was the model containing both traditional and next-generation RS variables (Table 2). At the coarse scale (210 m), fox squirrels preferred Grassland/Herbaceous, Pasture/Hay, and Woody Wetlands more and Shrub/Scrub, and they preferred Emergent Herbaceous Wetland and Developed/Open Space less than the reference class Evergreen Forest; fox squirrels also selected areas with greater tree cover. Fox squirrels selected against eastern aspects and areas with lower slopes. There was an inverse relationship between fox squirrel habitat selection and the values of CHM and the MSI. Fox squirrels selected areas with higher values of fPAR.

At the intermediate scale (30 m) of selection, all land cover classes except Mixed Forest were selected less than the reference class of Evergreen Forest, and areas with greater tree cover were preferred. Fox squirrels were selected for more southern aspects and greater slopes. Finally, greater values of CHMs and LAIs increased the probability of use by fox squirrels. The best intermediate-scale model had about four times less explanatory power than the best coarse-scale model.

At the fine spatial scale (10 m), fox squirrel use was positively associated with more southern aspects and greater slopes. Higher values of CHMs and MSIs and lower values in fPAR increased the probability of fox squirrel use. The best fine-scale model had nearly three times more explanatory power than the best intermediate-scale model, but it was still less than the best coarse-scale model.

### 3.2. Evaluation of the Multi-Scale Model

Of models using traditional and next-generation variables, the multi-scale model was by far the most parsimonious, and this model also had the most explanatory power (Table 3). Therefore, we used the multi-scaled model to describe fox squirrels’ predicted response to environmental features at different scales. 

**Figure 2:**
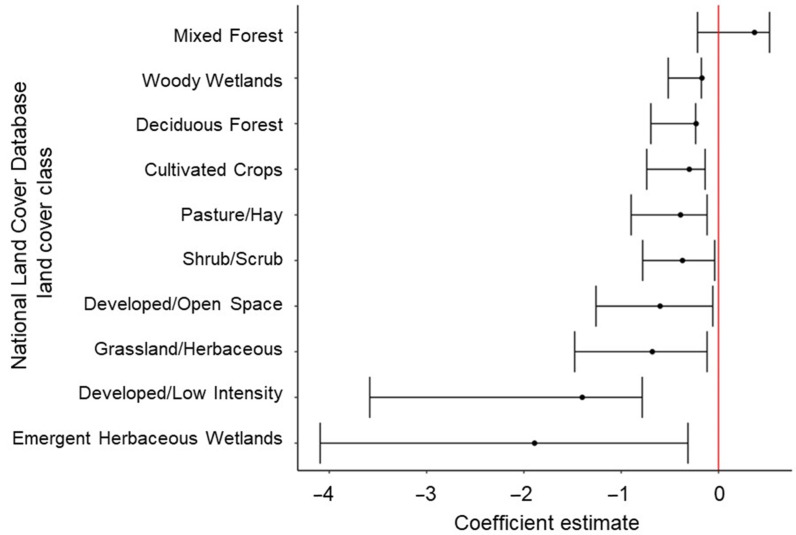
Modeled estimates with 95% confidence intervals regarding fox squirrel (*Sciurus niger*) habitat selections using the National Land Cover Database land cover classes compared to the reference category of Evergreen Forest at 30 m. Fox squirrel locational data (*n* = 82,012) was collected at the Jones Center at Ichauway in Georgia and the Ordway-Swisher Biological Station in Florida in 2016 and 2017. Red-line illustrates 95% CIs relationship with 0.

**Figure 3:**
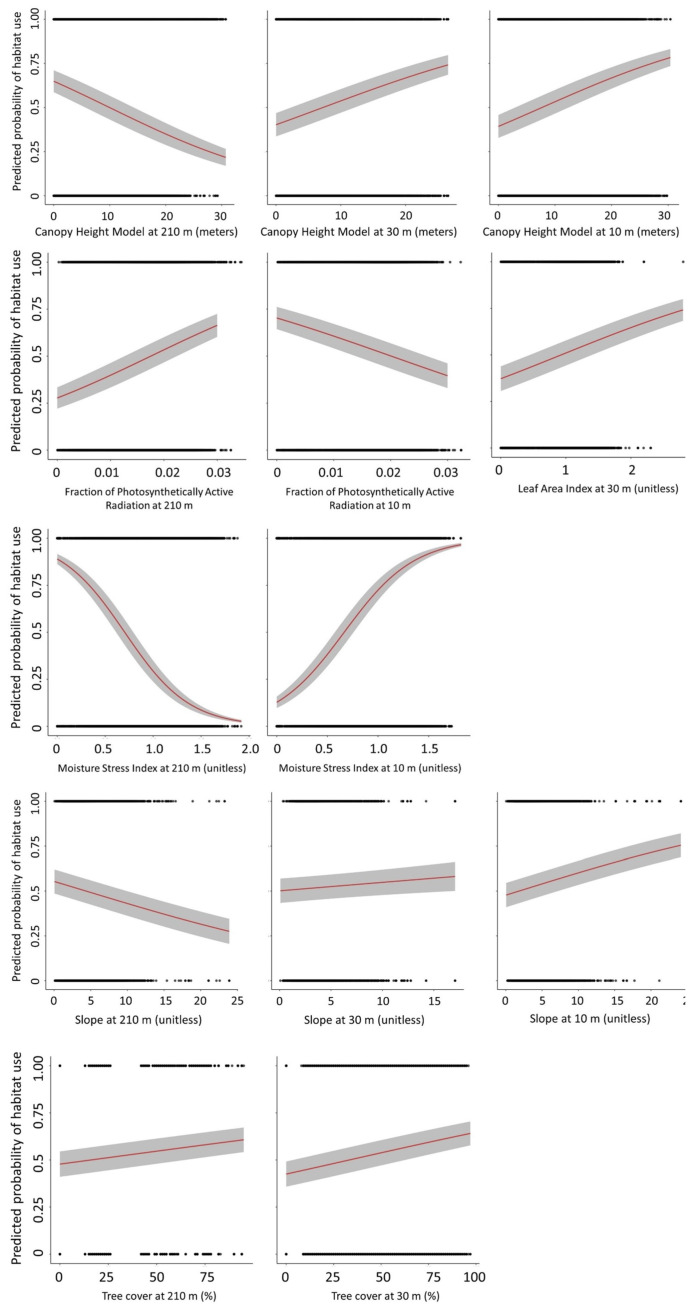
Modeled estimates (red line) with 95% confidence intervals (gray area) and values (black dots) for significant remotely sensed variables from the multi-scale generalized linear mixed model of the proportion of GPS habitat locations (*n* = 82,012) that were used by fox squirrels (*Sciurus niger*) at the Jones Center at Ichauway in Georgia and the Ordway-Swisher Biological Station in Florida in 2016 and 2017. Variables were assessed at 210 m, 30 m, and 10 m scales and included a Canopy Height Model, a fraction of photosynthetically active radiation (fPAR), a Leaf Area Index (LAI), Moisture Stress Index, slope, and tree cover.

**Figure 4:**
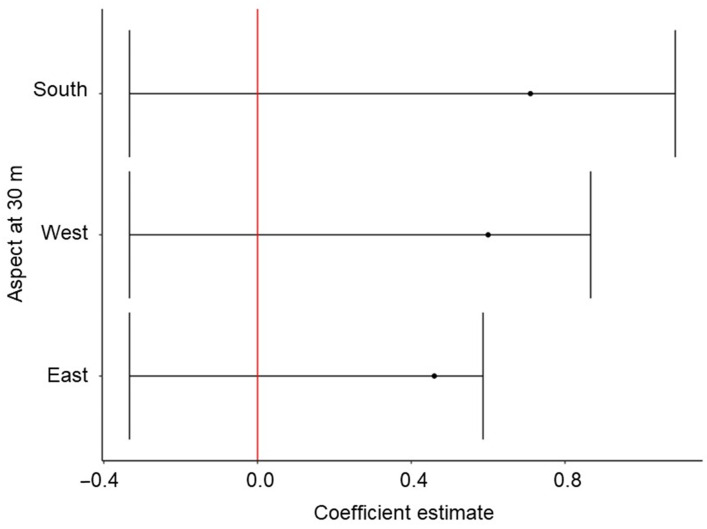
Modeled estimates with 95% confidence intervals of fox squirrels (*Sciurus niger*) habitat selections) of aspect compared to the reference category of North at 30 m. Fox squirrel locational data (*n* = 82,012) was collected at the Jones Center at Ichauway in Georgia and the Ordway-Swisher Biological Station in Florida in 2016 and 2017. Red-line illustrates 95% CIs relationship with 0.

Coefficients associated with most land cover classes at 210 m and at 30 m differed from the reference category of Evergreen Forest (Table 4). Similar to the best single-scale model at 210 m, Grassland/Herbaceous and Pasture/Hay at 210 m were more preferred than Evergreen Forest. At 30 m, only Mixed Forest was more preferred than Evergreen Forest (Figure 2). The predicted probability of habitat use based on slope decreased approximately 5% for five units of change at 210 m but increased by approximately 3% for five units of change at 30 m and 5% for five units of change at 10 m (Figure 3). The CHM variable at the 210-m scale affected fox squirrel habitat use, decreasing the predicted habitat use by approximately 12% for every 10 m of canopy height (Figure 3). The CHM variable at 30- and 10-m scales also affected fox squirrel habitat use, increasing predicted habitat use by approximately 10% at both scales for every 10 m of canopy height. Fox squirrel habitat use increased by approximately 5% for every 20% of increase in tree cover at 210 m and was slightly greater than 5% for every 20% of increase in tree cover at the 30-m scale (Figure 3). In regard to the aspect at 210 m, fox squirrel habitat use was most associated with western aspects, yet at 30 m (Figure 4) and at 10 m, fox squirrel habitat use was most associated with southern aspects.

Based on the relative strength of modeled Beta estimates, fox squirrels’ selection for MSI at 210 m and 10 m was the most prominent among all explanatory variables (Table 4). At the 210 m scale, the predicted probability of fox squirrel use decreased by approximately 90% over the range of observed values. Alternatively, we saw an increasing probability of use over the range of observed values at 10 m that was almost as pronounced (Figure 3). At the 210 m scale, fPAR increased the probability of fox squirrel use almost three times over the range of the values observed, while at the 10 m scale, the probability of fox squirrel use decreased by approximately two times over the range of observed fPAR values (Figure 3). At the 30 m scale, the probability of use increased approximately two times over the range of observed LAI values observed (Figure 3).

## 4. Discussion

We found that both next-generation RS variables and examination of multiple spatial scales improved our ability to predict the habitat selection of a small mammal, the fox squirrel. Our multi-scale model was by far more parsimonious than any single-scale model and outperformed single-scale models’ explanatory power by more than 10%. In our multi-scale model, two next-generation RS measures of vegetation at fine spatial scales (plant moisture stress (MSI) and fraction of photosynthetically active radiation (fPAR)) were important predictors of fox squirrel habitat selection. These vegetation variables, while more commonly measured for larger species [4], are rarely used to understand small mammals’ space use [1,39] but collectively suggest the importance of understory vegetation to fox squirrels.

Fox squirrel habitat selection has been studied extensively in the southeastern United States. Researchers have used fine spatial scale variables in some of these studies, and these variables were measured in the field [12,15,35], requiring additional labor, financial resources, and potentially disturbing study animals’ behavior. We used the results of prior studies to identify potentially useful next-generation RS variables for our study (Table 1). Our resource selection models agreed with the findings and predictions regarding overstory structures resulting from prior studies and offered further insight into the importance of an herbaceous understory. Management recommendations from our resource selection study agree with those of others [15], suggesting the importance of silvicultural practices and regular prescribed fires that maintain open-canopied pine forests comprised of large trees and dense herbaceous understories. Our research, however, did not require intensive on-the-ground sampling.

Fox squirrels’ movements were strongly linked to elevated plant moisture stress at fine spatial scales (Table 4). We suspect that this is because the MSI is related to a reduction in understory vegetation [40], which fox squirrels seem to prefer [35]. Additionally, fox squirrels could be associated with reduced moisture because pine trees may produce more cones if conditions are dry during certain months in the first two years of their growth cycle [41]. Further, production of acorns in some oak species, another important food resource for fox squirrels, has been negatively correlated with precipitation in previous years [42].

The fPAR variable describes the absorption of light (400–700 nm) by vegetation, is related to primary productivity, and can be used as an indicator of vegetation health. Fox squirrels’ habitat selection was negatively associated with fPAR at fine spatial scales. Like the positive relationship between fox squirrel use and MSI, we suspect fox squirrels may select areas of open canopy that generally have less vegetation productivity and biomass than closed canopy forests. Although fPAR has rarely been used in small-mammal studies [39], it has been used to identify refuges from fire and drought [43]. More research is needed to elucidate why fox squirrels may select habitats with lower fPAR and higher MSI values at fine spatial scales. Importantly, our multi-scale model was clearly our most parsimonious model; there were no competing models (the next lowest delta AICc was 12,978). If competing models exist, researchers should consider model averaging to reduce the risk of overfitting [36].

By using variables that encompassed habitat structure, land cover, topography, and vegetation physiology in our multi-scale models, we found that fox squirrels select for a suite of variables represented by RS metrics at all examined spatial scales. As expected, our multi-scale model was more informative than any single-scale model because fox squirrels selected different variables at different spatial scales. However, aspect, slope, and tree height (from CHM) were important at all spatial scales.

At the broadest scale (210 m), land cover and tree cover were important for fox squirrel habitat selection. In contrast to previous studies, at a similar scale (5.3 ha; [20]), fox squirrels preferred areas with increased tree cover. This was likely because our study sites were centered on open-canopy pine savannahs and not a random sample of forest cover types [20]. Also, fox squirrels seemed to prefer some land cover types that were rare on our study sites, but these cover types may not have been sufficiently available to quantify their importance to fox squirrels. Importantly, our research suggests aspect and three next-generation variables—CHM, fPAR, and MSI—as important predictors of habitat use at broad scales.

On the intermediate scale (30 m), our results were similar to those that found that fox squirrels were associated with Mixed Forest [21]. Our research adds aspect, tree height (from CHM), LAI, tree cover, and slope as important predictors of habitat use at this scale. Importantly, we found that this spatial scale was the least predictive (e.g., AICc and Beta Estimates; Table 3 and Table 4) despite being the most common spatial scale in terms of using RS data to model wildlife habitats. At fine scales, in addition to MSI and fPAR, the CHM was a significant predictor of fox squirrel use, emphasizing the importance of large trees for fox squirrels [14,15,25]. Our research also suggests aspect and slope as predictors of habitat use at a fine scale.

Here, we show that ecologists can improve our understanding of small-animal–environment relationships by coupling GPS technology with modern RS data. We expect that the ability to better understand small-animal habitats will continue to improve with further technological developments. For example, we were unable to fully match the grain of our datasets due to limitations of the location accuracy of our GPS tracking equipment. Improvements in tracking technology accuracy are now needed to match the resolution of currently available fine-scale RS data. When tracking technology becomes more accurate vertically, i.e., elevation, LiDAR may become particularly useful for understanding the habitat selection of animals that use tree canopies.

Although we could not fully match the grain of animal location and next-generation RS data, we suggest that the spatial resolution between datasets is much more comparable than in the past. We caution, however, that the temporal resolution of next-generation RS data may be incompatible with small mammal movement data. Our study animals were tracked over approximately two years, but the next-generation RS data were collected in one month. Lack of simultaneously collected RS and animal movement data may reduce the utility of some next-generation RS data as predictors of animal habitat selection. In our analyses, we suggest that this was not the case.

We were able to interpret next-generation RS predictors in ways that were less sensitive to potential temporal variations. For example, we interpreted fox squirrel selection based on MSI and fPAR—two RS variables that are expected to vary temporally—as being due to squirrels selecting open-canopied pine forests with herbaceous ground cover. In this case, these variables were used to make inferences regarding vegetation structure as opposed to function. In other words, we used relative values to ascertain structural features at the time of data collection as opposed to focusing on functional variations across space or time. Moreover, we had a priori expectations that fPAR [14] and MSI [35] may be useful predictors of fox squirrel habitat selection.

Also, pixel-based RS data such as ours is likely to be less informative than the next wave of RS-derived products, such as NEON-derived individual tree [44] and shrub maps. Satellite RS platforms with very high spatial resolution, which are necessary to create such products on a global scale, have not yet been developed but hold promise for the future. These products will be extremely useful because small mammals respond to environmental boundaries [45] that are more readily detected at high spatial resolutions due to fewer mixed pixels, which can hinder interpretation of vegetation data and introduce errors into habitat models. Finally, future research should examine the usefulness of next-generation RS data in developing habitat models for other small animals to determine whether these variables provide an increased understanding of habitat selection in other animals.

Although our next-generation RS products were obtained from NEON, satellite-based RS data with the ability to create similar RS products (e.g., MSI and fPAR) are currently available. The Sentinel-2C satellite, part of the European Space Agency Copernicus application, was launched in 2024. This satellite can collect imagery at a 10 m spatial resolution and at bandwidths that will allow for the development of data products similar to those used in our research. Further, unlike NEON data, Sentinel-2C data have a five-day return interval [46].

## 5. Conclusions

We discovered important relationships between fox squirrels’ use of space and the RS metrics of vegetation physiology that were estimated across large areas at fine resolutions. At all the spatial scales examined, the inclusion of next-generation RS variables improved the explanatory power of the models. Like previous studies [15,20,30], we showed the importance of addressing multiple spatial scales when studying small-mammal habitat associations (i.e., multi-scale models). We caution that multi-scale models can be difficult to interpret, especially when signs of coefficients differ (i.e., positive and negative coefficients for slopes) among spatial scales, as with what occurred in our multi-scale model. However, differing slopes should be expected when different resources are selected among scales [29]; for example, a home range may be selected for breeding opportunities, but, within the home range, selection may occur based on foraging opportunities. Our approach generated a more complete understanding of fox squirrel selection of habitats without requiring on-the-ground vegetation sampling [15,20,30]. Our results can be applied to better manage fox squirrels [19,47] and show how new technologies can advance our understanding of small mammals while reducing the need for sampling vegetation in the field.

## Figures and Tables

**Table 2:** Beta coefficients (Standard Error) of single-scale generalized linear mixed models of the proportion of GPS habitat locations (*n* = 82,012) that were used by fox squirrels (*Sciurus niger*) at the Jones Center at Ichauway in Georgia and the Ordway-Swisher Biological Station in Florida in 2016 and 2017 as a function of traditional and next-generation remotely sensed environmental variables at broad (210 m), intermediate (30 m), or fine (10 m) spatial scales.

	210 m		30 m		10 m	
Effects	Traditional	Traditional + Next-Generation	Traditional	Traditional + Next-Generation	Traditional	Traditional + Next-Generation
Intercept	−0.50 (0.11) ^a,^***	−0.08 (0.14)	−0.69 (0.16) ***	−0.44 (0.16) **	−0.03 (0.06)	−0.73 (0.12) ***
Study site	0.42 (0.16) **	1.04 (0.21) ***	−0.43 (0.10) ***	−0.56 (0.12) ***	−0.01 (0.02)	−0.36 (0.15) *
Aspect						
North ^a^						
East	0.48 (0.06) ***	−0.15 (0.06) *	0.75 (0.14) ***	0.53 (0.14) ***	0.04 (0.07)	0.77 (0.07) ***
South	0.60 (0.06) ***	−0.11 (0.06)	1.07 (0.14) ***	0.83 (0.14) ***	0.02 (0.06)	0.94 (0.07) ***
West	0.57 (0.06) ***	0.12 (0.06)	0.80 (0.14) ***	0.60 (0.14) ***	0.07 (0.06)	0.69 (0.07) ***
CHM		−0.31 (0.01) ***		0.24 (0.01) ***		0.39 (0.01) ***
fPAR		0.20 (0.01) ***				−0.18 (0.01) ***
Land cover						
Developed, Open Space	−1.12 (0.06) ***	−1.05 (0.07) ***	−0.63 (0.03) ***	−0.63 (0.03) ***		
Developed, Low Intensity	N/A	N/A	−1.63 (0.33) ***	−1.60 (0.33) ***		
Deciduous Forest	18.67 (795.2)	16.62 (635)	−0.18 (0.10)	−0.17 (0.10)		
Evergreen Forest ^a^						
Mixed Forest	17.46 (1179)	15.26 (1159)	−0.02 (0.10)	−0.01 (0.10)		
Shrub/Scrub	−0.45 (0.02) ***	−0.14 (0.02) ***	−0.37 (0.02) ***	−0.37 (0.02) ***		
Grassland/ Herbaceous	0.78 (0.10) ***	0.96 (0.10) ***	−0.71 (0.05) ***	−0.72 (0.05) ***		
Pasture/Hay	0.07 (0.05)	0.24 (0.05) ***	−0.44 (0.05) ***	−0.45 (0.05) ***		
Cultivated Crops	19.38 (689.3)	16.96 (561)	−0.16 (0.06) *	−0.16 (0.06) *		
Woody Wetlands	0.99 (0.07) ***	0.30 (0.07) ***	−0.32 (0.07) ***	−0.35 (0.07) ***		
Emergent Herbaceous Wetlands	−2.67 (0.18) ***	−2.49 (0.19) ***	−1.87 (0.14) ***	−1.90 (0.14) ***		
LAI				0.14 (0.01) ***		
MSI		−1.27 (0.01) ***		0.02 (0.02)		1.33 (0.01) ***
Slope	0.10 (0.01) ***	−0.10 (0.01) ***	0.11 (0.01) ***	0.05 (0.01) ***	−0.03 (0.01) ***	0.15 (0.01) ***
Tree cover	0.04 (0.01) ***	0.17 (0.01) ***	0.37 (0.01) ***	0.20 (0.01) ***		
Log-likelihood	−53,290	−46,400	−54,694	−54,257	−56,835	−49,458
Delta AICc	13,775	0	867	0	14,748	0
Psuedo-*r*^2^	0.49	0.58	0.10	0.12	0.0003	0.31

Model coefficients were significant at α = 0.05 with *p*-values: * <0.05, ** <0.01, *** ≤0.001. Response variable: Location used by fox squirrel or random location. Fixed effects: Land cover = the mode of National Land Cover Database class aggregated to X m. Aspect = the mean of numerical aspect aggregated to X m, then categorized as north, east, south, or west. Biomass (BIOM), tree cover, Canopy Height Model (CHM), fraction of photosynthetically active radiation (fPAR), Leaf Area Index (LAI), Moisture Stress Index (MSI), Normalized Difference Vegetation Index (NDVI), and slope = the mean value of numerical data layers at X m, where X = 210, 30, or 10. Random effect: 1|Animal = to account for variation in individual squirrels. ^a^ = reference categories. N/A = cover type does not exist at this scale. ^a^ Reference category of categorical variables.

**Table 3:** Model selection results of best single-scale and the multi-scale habitat selection models for fox squirrels (*Sciurus niger*) at the Jones Center at Ichauway in Georgia and the Ordway-Swisher Biological Station in Florida in 2016 and 2017 as a function of traditional and next-generation remotely sensed environmental variables at broad (210 m), intermediate (30 m), and/or fine (10 m) spatial scales.

Model Type	Spatial Scale (m)	Remotely Sensed Variable Type (s)	Delta AICc	Pseudo-*r*^2^
Multi-scale model	210 + 30 + 10	Traditional + Next-generation	0	0.68
Single-scale model	210	Traditional + Next-generation	12,978	0.58
Single-scale model	10	Traditional + Next-generation	19,074	0.31
Single-scale model	30	Traditional + Next-generation	28,695	0.12

**Table 4:** Beta coefficients (Standard Error) of the multi-scale generalized linear mixed model of the proportion of GPS habitat locations (*n* = 82,012) that were used by fox squirrels (*Sciurus niger*) at the Jones Center at Ichauway in Georgia and the Ordway-Swisher Biological Station in Florida in 2016 and 2017 as a function of traditional and next-generation remotely sensed environmental variables at broad (210 m), intermediate (30 m), and fine (10 m) spatial scales.

Effects		210 m	30 m	10 m
Intercept	−1.24 (0.27) ^a,^***			
Study site	0.01 (0.03)			
Aspect				
North ^a^				
East		−0.19 (0.07) **	0.46 (0.17) **	0.87 (0.07) ***
South		−0.12 (0.07)	0.71 (0.17) ***	1.01 (0.07) ***
West		0.19 (0.07) **	0.60 (0.17) ***	0.84 (0.08) ***
CHM		−0.37 (0.01) ***	0.23 (0.01) ***	0.31 (0.01) ***
fPAR		0.22 (0.01) ***		−0.16 (0.01) ***
Land cover				
Developed, Open Space		−1.11 (0.07) ***	−0.60 (0.03) ***	
Developed, Low Intensity		N/A	−1.40 (0.40) ***	
Deciduous Forest		17.83 (1121)	−0.23 (0.12)	
Evergreen Forest ^a^				
Mixed Forest		15.46 (1192)	0.37 (0.11) **	
Shrub/Scrub		−0.14 (0.02) ***	−0.37 (0.02) ***	
Grassland/Herbaceous		0.71 (0.11) ***	−0.68 (0.06) ***	
Pasture/Hay		0.26 (0.05) ***	−0.39 (0.06) ***	
Cultivated Crops		18.04 (1100)	−0.30 (0.07) ***	
Woody Wetlands		0.04 (0.08)	−0.17 (0.09) *	
Emergent Herbaceous Wetlands		−2.28 (0.20) ***	−1.89 (0.16) ***	
LAI			0.15 (0.01) ***	
MSI		−1.22 (0.01) ***		1.18 (0.01) ***
Slope		−0.08 (0.01) ***	0.06 (0.01) ***	0.12 (0.01) ***
Tree cover		0.15 (0.01) ***	0.20 (0.01) ***	
Log-likelihood	−39,887			
Pseudo-*r*^2^	0.68			

Model coefficients were significant at α = 0.05. *p*-values: * <0.05, ** <0.01, *** ≤0.001. Response variable: location used by fox squirrel or random location. Fixed effects: Land cover_X = the mode of National Land Cover Database class aggregated to X m. Aspect = the mean of numerical aspect aggregated to X m then categorized as north, east, south, or west. Tree cover, Canopy Height Model (CHM), fraction of photosynthetically active radiation (fPAR), Leaf Area Index (LAI), Moisture Stress Index (MSI), slope = the mean value of numerical data layers at X m, where X = 210, 30, or 10. Random effect: 1|Animal = to account for variation in individual squirrels. ^a^ Reference category of categorical variables.

## Data Availability

The original data and code are available on FigShare https://figshare.com/s/d278507e5f1855c7af03, accessed on 17 October 2024.
